# Correlation between mortality and blood transfusion in patients with major surgery initially admitted to intensive care unit: a retrospective analysis

**DOI:** 10.1186/s12871-023-02261-3

**Published:** 2023-09-04

**Authors:** Hua Xiao, Wei Song, Hongmei Ai, Jingpeng Zhang, Jing Lu, Danping Zhang, Zaiwen Zhou, Pu Xu

**Affiliations:** 1 Department of Blood Transfusion, Huanggang Central Hospital of Yangtze University, Huanggang, 438000 China; 2 Department of Blood Transfusion, Jingzhou Hospital Affiliated to Yangtze University, Jingzhou, 434000 China; 3 Department of Critical Care Medicine, Huanggang Central Hospital of Yangtze University, Huanggang, 438000 China; 4 Department of Blood Transfusion, The People’s Hospital of Tuanfeng, Tuanfeng, 438800 China; 5 Department of Blood Transfusion, Renmin Hospital of Wuhan University, Wuhan, 430060 China

**Keywords:** Intensive care unit, Major surgical procedure, Fatality rate, Blood transfusion

## Abstract

**Purpose:**

Transfusing red blood cells promptly corrects anemia and improves tissue oxygenation in around 40% of patients hospitalized in the intensive care unit (ICU) after major surgical operations. This study’s goal is to investigate how blood transfusions affect the mortality rates of patients after major surgery who are hospitalized in the ICU.

**Methods:**

Retrospective research was done on recently hospitalized patients who had major procedures in the ICU between October 2020 and February 2022 at the Huanggang Central Hospital of Yangtze University, China. The patients’ prognoses at three months were used to classify them as either survivors or deceased. Patient demographic information, laboratory results, and blood transfusion histories were acquired, and the outcomes of the two groups were compared based on the differences. Univariate and multivariate logistic regression analyses were used to examine the prognosis of surgical disease patients first admitted to the ICU. The receiver operating characteristic (ROC) curve was used to evaluate the predictive power of each risk factor. The relationship between transfusion frequency, transfusion modality, and patient outcome was examined using Spearman’s correlation analysis.

**Results:**

Data from 384 patients was included in the research; of them, 214 (or 55.7%) died within three months of their first stay in the ICU. The death group had higher scores on the Acute Physiology and Chronic Health Evaluation II (APACHE II) and the Sequential Organ Failure Assessment (SOFA) than the survival group did (all P < 0.05); the death group also had lower scores on the Glasgow Coma Scale, systolic blood pressure, hemoglobin, platelet distribution width, and blood transfusion ratio. Multivariate logistic regression analysis revealed an odds ratio (OR) of 1.654 (1.281–1.989), a 95% confidence interval (CI) of 1.440 (1.207–1.701), and a P value of 0.05 for death in patients undergoing major surgery who were hospitalized to the intensive care unit (ICU). Areas under the ROC curve (AUC) of 0.836, 0.799, and 0.871, respectively, and 95% CIs of 0.796–0.875, 0.755–0.842, and 0.837–0.904, respectively, all P0.05, had significant predictive value for patients initially admitted to the ICU and for APACHE II score > = 12 points, SOFA score > = 6, and blood transfusion. When all three indicators were used jointly to predict a patient’s prognosis after major surgery, the accuracy increased to 86.4% (sensitivity) and 100% (specificity). There was a negative correlation between the number of blood transfusions a patient had and their outcome (r = 0.605, P < 0.001) and death (r = 0.698, P < 0.001).

**Conclusion:**

A higher initial ICU APACHE II score, SOFA score, and a number of blood transfusions were associated with improved survival for patients undergoing major surgical operations. Patients’ death rates have increased with the increase in the frequency and variety of blood transfusions.

## Introduction

Patients who have had significant surgery or a traumatic accident and need round-the-clock monitoring of their vital signs are often admitted to the intensive care unit (ICU) [1, 2]. There is a significant death rate among ICU patients who suffer from a wide range of underlying disorders and pathological processes. C-reactive protein (CRP) levels, the Glasgow coma scale (GCS), the Sequential Organ Failure Assessment (SOFA), and the Acute Physiology and Chronic Health Evaluation II (APACHE II) score may all be used to evaluate a patient’s condition [3–5]. Meanwhile, the capillary refill time is also evaluated as a factor in assessing short-term mortality rates in patients. Researchers such as Mrgan et al [6]. found a significant correlation between CRT and short-term mortality through a prospective cohort study. Therefore, it should be advocated as a rapid tool for assessing circulatory status. The different collection sites may also affect accuracy and precision [7]. When treating severely ill patients, blood transfusions are often carried out, particularly for individuals who have been hospitalized in the ICU due to major surgical procedures. Vigilant monitoring and meticulous assessment of fluid responsiveness are equally crucial, and electronic evaluation is highly important. Two studies conducted by Monnet et al [8, 9]. indicate that passive leg raising reliably predicts changes in cardiac output and arterial pressure, serving as a robust predictor of adult volume changes. Additionally, the introduction of artificial intelligence with automated boundary detection capabilities enhances the efficacy in predicting changes in inferior vena cava dynamics, thus reflecting fluid responsiveness more effectively [10]. Chronic conditions such as sleep apnea can lead to anaemia over time. For example, sleep apnoea usually occurs in conjunction with conditions such as obesity, diabetes and high blood pressure, which can affect the production or survival of red blood cells and lead to anaemia [11]. In addition, acute blood loss, phlebotomy, and decreased erythropoiesis can lead to hypovolemia. Therefore, a timely and adequate blood supply is important in the rescue process.

Rapid correction of anemia and improved tissue oxygenation is achieved with red blood cell infusions in around 40% of patients admitted to the ICU after major surgical operations. Blood transfusions are defined as the administration of whole blood or blood components to a patient when they are in the ICU, as stated by the applicable guidelines of the People’s Republic of China Health Industry Standard WS/T623-2018 “Use of Whole Blood and Blood Components.“. Patients’ survival rates may be enhanced via blood transfusions since they assist in raising total blood volume, hasten the return of normal vital signs, speed up the delivery of oxygen to tissues, and restore blood pressure and heart rates. Patients with major surgery have a mortality rate of 17–70%, according to various studies, even after receiving large blood transfusion treatment [12, 13]. The association between mortality and blood transfusion in patients undergoing major surgery who are first brought to the ICU is currently the subject of few investigations. Therefore, the goal of this study was to evaluate the relationship between mortality rates and blood transfusions in patients who were hospitalized in the ICU following major procedures. Our study’s main goal is to determine the optimum combination between the advantages and hazards of blood transfusion treatment in the context of large-scale blood transfusion, which will be utilized as a benchmark for reducing mortality rates.

## Materials and methods

### Ethical approval and patients

This retrospective study was approved by the Institutional Review Board of Huanggang Central Hospital, Yangtze University, China (IRB No. 202,004,106), and the study complied with the Helsinki Declaration and local laws. This study was conducted on newly admitted patients between October 2020 and February 2022 after major surgeries in the ICU of the Huanggang Central Hospital of Yangtze University, China. Subject data were included if they met the following criteria: Patients had to be hospitalized in the ICU because of: 1 Major cardiac, thoracic, general, neurological, tumor, or urological surgery; 2 All patient case data was complete. The factors that led to the data’s exclusion were as follows: Patients with severe coagulation abnormalities; patients who were treated with anticoagulant medicines during the previous 15 days; patients who had an Abbreviated Injury Scale (AIS) score of 3 at the time of admission.

### Grouping of research subjects

The participant data who had been followed up for a total of three months were included in the study. Three-month survival rates were used to categorize the patients into either a survival group or a death group.

### Collection of patients’ data

① Patient background data and laboratory test results: All the Patients’ information including demographic, illness, APACHE II score, GCS, SOFA score, heart rate, body temperature, systolic blood pressure, and transfusion history were reviewed and noted. Regular blood indicators such as hemoglobin, platelet volume distribution width, platelet count, neutrophil-to-lymphocyte ratio, and platelet-to-lymphocyte ratio were collected. In addition, Coagulation function indicators, including prothrombin time, international normalized ratio, and activated partial thromboplastin time, and inflammatory markers, including C-reactive protein, serum amyloid A, and procalcitonin, were also collected. The number and type of blood transfusions used for the patients were noted from the patients’ data. The survival rates of all patients were counted after they were monitored for three months following their first admission to the ICU.

### Statistical methods

All statistical analyses were performed in SPSS (version 22.0). Since the data followed a normal distribution, we could utilize mean ± SD to do t-test group comparisons. The enumeration results were expressed as percentages, and the Chi-square (χ2) test was used to do between-group comparisons. Patients who underwent surgeries and were initially admitted to the intensive care unit were analyzed using a multivariate logistic regression model, which took into account only the indicators that showed significant differences in the univariate study and left out the independent risk variables impacting prognosis. Using a receiver operator characteristic curve (ROC), we evaluated how well each risk factor predicted the patient’s prognosis. Spearman’s correlation analysis was used to examine the association between blood transfusion volume and patient outcomes. The p-value for significance was determined to be < 0.05.

## Results

### Comparison of general data, laboratory parameters, and blood transfusion between the two groups of patients

Table 1 displays the demographics, laboratory measurements, and blood-transfusion history of both patient groups. There were 384 patients, 212 males, and 172 women, ranging in age from 25 to 74 (mean = 51 ± 6.4) years old. Of the total sample size of 384, 214 (55.73%) were classified as “died” due to passing away within three months of their initial ICU stay. Higher APACHE II and SOFA scores, lower Glasgow Coma Scale, systolic blood pressure, hemoglobin, packed cell volume, and blood transfusion ratio, higher prothrombin time (PT), international normalized ratio (INR), and activated partial thromboplastin time (APTT) values were observed in the death group when compared to the survival group (all P < 0.05, Table 1). Table 1 shows that neither group differed significantly from the other in terms of gender, age, illness, NLR, PLR, CRP, SAA, or PCT.

**Table 1:** Comparison of the two groups with varied prognoses of general information, laboratory results, and blood transfusion in patients with surgical diseases who were originally brought to the ICU.

Groups	Death group	Survival group	*χ*^*2*^/*t* value	*P* value
**n**	214	170		
**Gender (n)**			1.463	0.226
Male	90	82		
Female	124	88		
**Age (years, mean ± SD)**	51.2 ± 7.2	51.1 ± 6.9	0.245	0.738
**Disease type [n (%)]**			0.871	0.663
After cardiac surgery	42 (19.6)	30 (17.6)		
Severe trauma	28 (13.1)	24 (14.1)		
After thoracic surgery	28 (13.1)	20 (11.8)		
After general surgery	20 (9.3)	20 (11.8)		
After neurosurgery	30 (14.0)	22 (12.9)		
After tumor surgery	26 (12.1)	20 (11.8)		
After urology surgery	22 (10.3)	18 (10.6)		
After orthopedic surgery	10 (4.7)	8 (4.7)		
Other trauma	8 (3.7)	8 (4.7)		
**APACHE II score (points, mean ± SD)**	17.1 ± 4.6	10.6 ± 2.8	24.874	< 0.001
**GCS score (points, mean ± SD)**	8.0 ± 2.8	11.5 ± 3.4	-8.602	< 0.001
**SOFA score (points, mean ± SD)**	8.4 ± 1.2	4.3 ± 1.4	16.093	° 0.001
**Temperature (°, mean ± SD)**	36.1 ± 4.1	36.2 ± 5.2	1.387	0.531
**Heart rate (beats/min, mean ± SD)**	112.0 ± 7.4	111.8 ± 8.8	1.005	0.609
**Systolic blood pressure (mmHg, mean ± SD)**	82.2 ± 9.11	95.6 ± 8.5	-9.872	< 0.001
**PLT (10** ^**9**^ **/L, mean ± SD)**	112.8 ± 20.5	113.0 ± 21.2	-0.941	0.884
**Hb (g/L, mean ± SD)**	51.1 ± 8.2	69.2 ± 3.7	-18.957	< 0.001
**PDW (%, mean ± SD)**	9.5 ± 2.2	13.1 ± 2.3	-14.886	< 0.001
**NLR (mean ± SD)**	13.8 ± 3.3	14.0 ± 3.1	-2.038	0.104
**PLR (mean ± SD)**	220.1 ± 35.8	219.7 ± 41.1	1.751	0.226
**CRP (mg/L, mean ± SD)**	80.1 ± 19.3	79.9 ± 20.2	1.327	0.409
**SAA (mg/L, mean ± SD)**	298.1 ± 57.6	299.8 ± 60.0	-0.794	0.093
**PCT (µg/L, mean ± SD)**	1.1 ± 0.2	1.1 ± 0.3	0.035	0.088
**PT (s, mean ± SD)**	21.2 ± 3.9	15.0 ± 4.2	18.820	< 0.001
**INR (mean ± SD)**	1.8 ± 0.3	1.3 ± 0.3	15.997	< 0.001
**APTT (s, mean ± SD)**	45.0 ± 14.1	39.6 ± 12.7	13.295	< 0.001
**Blood transfusion [cases (%)]**			19.457	< 0.001
No	48 (22.4)	74 (43.5)		
Yes	166 (77.6)	96 (56.5)		

Abbreviations: APACHEII: acute physiology and chronic health valuation II; GCS: Glasgow coma scale; SOFA: sequential organ failure assessment; PLT: platelet; Hb: hemoglobin; PDW: platelet distribution width; NLR: neutrophil-to-lymphocyte ratio; PT: prothrombin time; PLR: platelet to lymphocyte ratio; SAA: serum amyloid A; PCT: procalcitonin; INR: international normalized ratio; CRP: C-reactive protein; APTT: activated partial thromboplastin time

### Univariate and multivariate logistic regression and ROC curve analysis

**Fig. 1:**
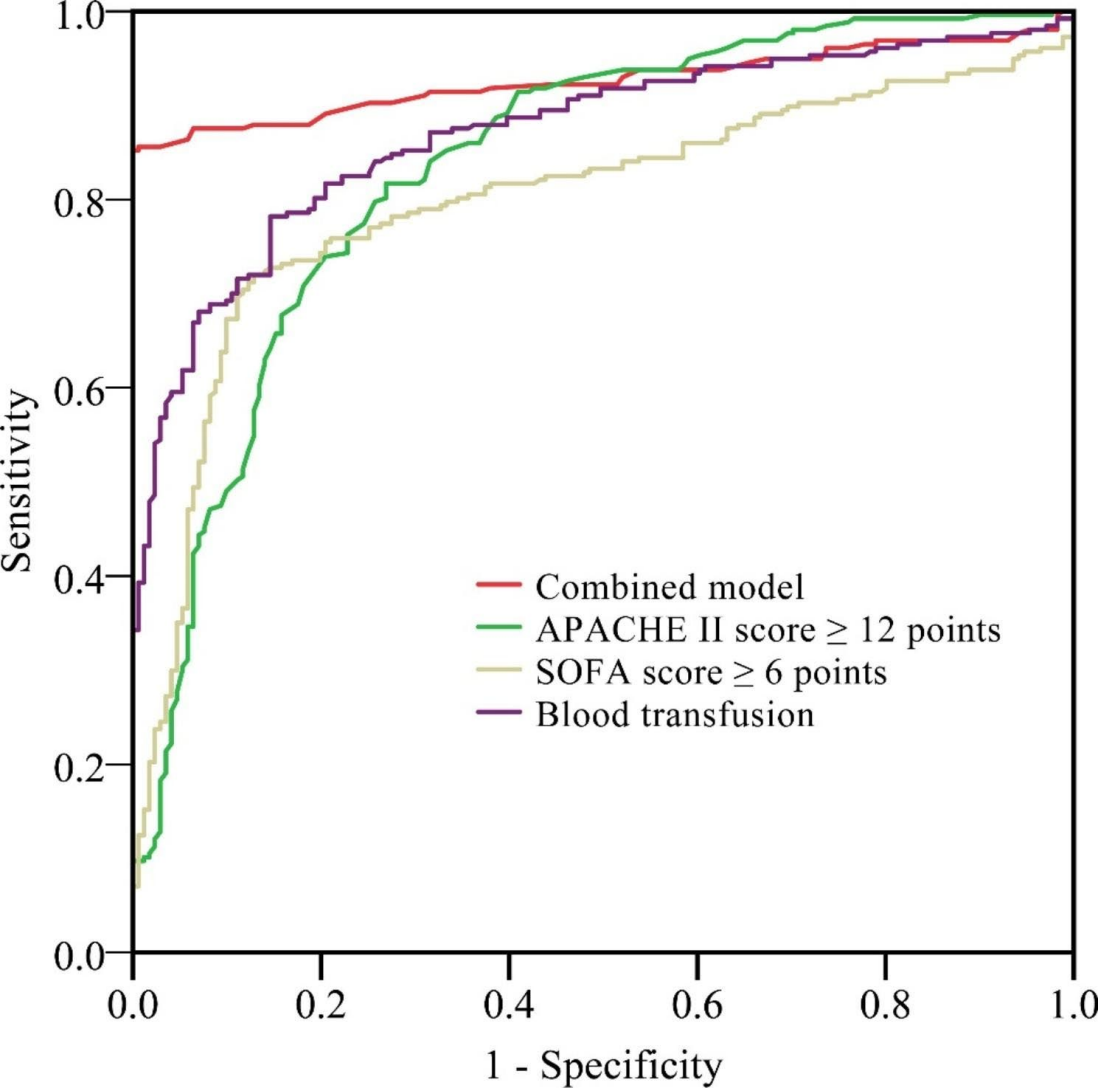
Analysis of the receiver operating characteristic (ROC) curve

The findings of a multivariate logistic regression analysis (Table 2) are shown after the indicators with statistically significant differences in the univariate analysis (Table 3) were assigned. Patients who had surgery before being admitted to the ICU had a poorer prognosis if they had an APACHE II score of ≥ 12 (β = 0.179, SE = 0.053, χ2 = 10.867, OR = 1.196 (95% CI: 1.074–1.329), p = 0.001), a Sequential Organ Failure Assessment score of 6 (β = 0.486, SE = 0.218, χ2 = 4.949, OR = 1.628 (95% CI: 1.057–2.509), p = 0.020), or a blood transfusion(β = 0.391, SE = 0.176, χ2 = 4.730, OR = 1.472 (95% CI: 1.041–2.078), p = 0.009) (all P < 0.05, multivariate logistic regression analysis). Patients first brought to the ICU with major surgery had a worse prognosis than those with other diagnoses (all P < 0.01, Table 4; Fig. 1) if their APACHE II score was ≥ 12 or higher, if their SOFA score was 6 or higher, or if they had had a blood transfusion.

**Table 2:** Univariate and multivariate logistic regression analysis on the prognosis of patients with surgical diseases initially admitted to the ICU

	Univariate model ^a^					Multivariate model ^a,b^				
Indicators	β	SE	*χ* ^*2*^	OR (95% CI)	*P*	β	Standardized β	*χ* ^*2*^	OR (95% CI)	*P*
APACHE II score ≥ 12 points	0.179	0.053	10.867	1.196 (1.074–1.329)	0.001	0.374	0.106	12.449	1.654 (1.281–1.989)	< 0.001
GCS score ≥ 9 points	0.024	0.126	0.038	1.024 (0.803–1.307)	0.868					
SOFA score ≥ 6 points	0.486	0.218	4.949	1.628 (1.057–2.509)	0.020	-0.362	0.198	7.301	1.440 (1.207–1.701)	0.002
Systolic blood pressure ≥ 90 mmHg	0.207	0.119	2.933	1.221 (0.979–1.548)	0.089					
Hb ≥ 60 g/L	-0.199	0.106	1.966	0.975 (0.949–0.998)	0.046	-0.021	0.098	1.387	0.994 (0.973–1.011)	0.299
PDW ≥ 10%	-0.003	0.184	0.081	0.976 (0.852–1.124)	0.472					
PT ≥ 13 s	0.199	0.041	9.063	1.219 (1.123–1.312)	0.003	0.008	0.168	0.963	1.012 (0.994–1.087)	0.395
INR ≥ 1.15	-0.105	0.026	5.068	0.897 (0.827–0.972)	0.008	0.016	0.273	1.267	0.994 0.986–1.005)	0.782
APTT ≥ 37 s	0.138	0.126	4.148	1.045 (1.025–1.674)	0.017	0.065	0.103	1.263	1.007 (0.989–1.073)	0.468
Blood transfusion	0.391	0.176	4.730	1.472 (1.041–2.078)	0.009	0.409	0.139	5.877	1.349 (1.039–1.755)	0.015

Abbreviations: OR: odds ratio; CI: confidence interval; APACHEII: acute physiology and chronic health valuation II; GCS: Glasgow coma scale; SOFA: sequential organ failure assessment; PDW: platelet distribution width; PT: prothrombin time; INR: international normalized ratio; APTT: activated partial thromboplastin time

^a^ Note that the odds ratio corresponds to a unit increase in the variable; ^b^ The odds ratio was adjusted for all significant variables of the univariate logistic regression analysis

**Table 3:** The assignment of factors affecting the prognosis of patients with surgical diseases in the ICU for the first time

Factors	Assignment
X1: APACHEII score	< 12 points = 0, ≥ 12 points = 1
X2: GCS score	< 9 points = 0, ≥ 9 points = 1
X3: SOFA score	< 6 points = 0, ≥ 6 points = 1
X4: Systolic blood pressure	< 90 mmHg = 0, ≥ 90 mmHg = 1
X5: Hb	< 60 g/L = 0, ≥ 60 g/L = 1
X6: PDW	< 10% = 0, ≥ 10% = 1
X7: PT	< 13 s = 0, ≥ 13 s = 1
X8: INR	< 1.15 = 0, ≥ 1.15 = 1
X9: APTT	< 37 s = 0, ≥ 37 s = 1
X10: Blood transfusion ratio	No blood transfusion = 0, blood transfusion = 1
Y: Survival situation	0 = alive, 1 = dead

Abbreviations: APACHEII: acute physiology and chronic health valuation II; GCS: Glasgow coma scale; PDW: platelet distribution width; INR: international normalized ratio; PT: prothrombin time; APTT: activated partial thromboplastin time; SOFA: sequential organ failure assessment

**Table 4:** The prognostic significance of each risk factor in patients with surgical disease receiving first ICU care

Risk factors	AUC	95%*CI*	*P* value	Sensitivity (%)	Specificity (%)	Youden index
APACHE II score ≥ 12 points	0.836	0.796–0.875	< 0.001	90.6	58.7	0.493
SOFA score ≥ 6 points	0.799	0.755–0.842	< 0.001	71.2	87.3	0.585
Blood transfusion	0.871	0.837–0.904	< 0.001	77.8	83.1	0.609
Combined model	0.926	0.899–0.954	< 0.001	86.4	100.0	0.864

Abbreviations: area under the receiver operating curve; CI: confidence interval; APACHEII: acute physiology and chronic health valuation II; SOFA: sequential organ failure assessment

### Number and type of blood transfusions correlate with surgical ICU patients’ prognosis

Patient outcomes were inversely linked with both the frequency and type of blood transfusions (r = 0.605, P < 0.001) and transfusions overall (r = 0.698, P < 0.001). The patient death rate increased as the frequency and variety of blood transfusions increased. The mortality rates of patients with less than 2 blood transfusions, 2 to 5 blood transfusions, and more than 5 blood transfusions were 20.6% (44/214), 34.6% (74/214), and 44.8% (96/214), respectively, (*P* < 0.05). Moreover, with infusion 1 or infusion 2 or infusion of 3 blood components, the mortality rate of patients was 22.4% (48/214), 33.7% (72/214), and 43.9% (94/214) (*P* < 0.05).

## 1 Discussion

Maintaining the blood oxygen supply to the tissue can ensure normal tissue cell metabolism and maintain normal organ function. However, for patients with major surgical procedures admitted to the ICU, owing to pathological reasons such as massive blood loss and occult bleeding, there are often pathological changes such as insufficient fibrinogen, thrombocytopenia, and shortened PT [14]. If patients do not receive timely and effective treatment, it can induce multiple organ failures and increase the risk of death. Therefore, timely blood transfusion therapy for patients with surgical diseases admitted to the ICU is of great significance for improving ischemia and hypoxia, promoting the recovery of hemorrhagic shock symptoms, and improving the treatment effect of patients. Blood transfusions have a broad and narrow scope. A narrow blood transfusion is one in which only whole blood is exchanged. Blood transfusion, in its broadest definition, encompasses not just whole blood transfusion but also transfusion of red blood cells, platelets, plasma, and any other physical or intangible component derived from blood. The transfusion of hematopoietic stem cells is also a special type of blood transfusion [15, 16]. The blood transfusion referred to in this study was a generalized blood transfusion. Patients with major surgical procedures who were admitted to the ICU for the first time mainly included those with severe trauma and those who required continuous and strict monitoring of vital indicators after major surgery. These patients mostly present with varying degrees of anemia, thrombocytopenia, and abnormal coagulation function. Therefore, it is often necessary to transfuse plasma, red blood cells, platelets, and other components or whole blood to improve anemia or coagulation dysfunction and relieve disease [17]. It was consistent with the findings of similar studies [18] that 214 of the 384 patients included in this analysis died within three months after their initial ICU hospitalization. We hypothesize that this may be due to patients in the intensive care unit often suffering from several pathological processes or illnesses, each of which contributes to their high fatality rate.

While the factors affecting the mortality of newly admitted surgical patients to the intensive care unit have been studied extensively, the association between blood transfusion and patient mortality has scarcely been studied. Patients with surgical procedures who were initially admitted to the ICU had a worse prognosis if they had an APACHE II score of 12 or higher, a SOFA score of 6 or higher, or had received a blood transfusion. Studies have shown that SOFA scores can effectively reflect organ dysfunction or failure in patients after major surgery and severe trauma. SOFA scores involve continuous variables and are characterized by a simple operation, easy access, and strong objectivity. At the same time, the variables used in the SOFA score were not related to race, disease type, or demographic characteristics, nor were they related to treatment measures. Furthermore, patients with major surgical procedures who are first brought to the ICU have a high mortality rate [19–21], and the SOFA score may be used to determine the extent of organ dysfunction and failure. Although the APACHE II score is also routinely used, it is heavily influenced by treatment considerations and should be used with caution when assessing the severity of an ICU patient’s condition. Consequently, the APACHE II score is only calculated for those admitted during the first 24 h, whether to a regular hospital room or an intensive care unit, whereas the SOFA score does not take into account the patient’s age or medical history. However, the score can be continuously assessed to reduce the influence of factors such as age, medical history, and illness [22].

The total amount of blood in the human body is approximately 8% of the body weight, and blood loss in patients after severe trauma or major surgery can exceed 30% of the peripheral blood volume. Therefore, hypovolemic shock is a common manifestation in patients with surgical diseases who are initially admitted to the ICU [23]. Hemorrhagic shock is mainly caused by a decrease in the body’s effective circulating blood volume and a decrease in tissue perfusion due to distal microcirculation disorders, resulting in tissue reperfusion injury [24]. After tissue reperfusion injury, the level of oxygen free radicals in the body can increase rapidly and accumulate in the body or cells, causing a decline in diffuse tissue perfusion and tissue oxidative damage. Therefore, timely replenishment of the blood volume is the main treatment method for such patients [25]. However, despite a greater transfusion rate, this research demonstrated that factors such as blood type and the number of transfusions did not affect survival. With the increase in the type and frequency of blood transfusions, the mortality rate of patients has also increased significantly. In the past, whole blood transfusion was mainly used for patients with hemorrhagic shock; however, supplementing a large amount of whole blood in the short term can increase the incidence of adverse blood transfusion reactions. In traditional blood transfusion, whole blood is uniformly transfused, regardless of the blood components that the patient needs to supplement. With the development of clinical medicine, immunology, biochemistry, and other disciplines, and in-depth research on blood components and functions, blood component transfusion has gradually replaced whole blood transfusion therapy. Transfusion of blood components not only improves the utilization rate of blood sources but also reduces the amount of blood transfusion and the cardiovascular burden of patients [26].Transfusing red blood cells can alleviate anemia and enhance tissue oxygenation, a strategy effective for around 40% of postoperative or sleep apnea patients in the intensive care unit. While robot-assisted surgeries might induce anemia due to blood loss, they generally involve lesser blood loss compared to traditional methods, potentially reducing the need for transfusions. However, for sleep apnea patients, heightened anesthesia-related risks necessitate careful management during any surgery, including robot-assisted procedures [27].

Patients with major surgical procedures who were admitted to the ICU for the first time often had massive bleeding. In addition to decreased blood volume, it is also easy to reduce the consumption of coagulation factors owing to massive blood loss, resulting in coagulation dysfunction and thrombocytopenia in the body. Therefore, a combined transfusion of two or three components of plasma, platelets, and red blood cells is often used. However, the number of blood transfusions and the more types of blood transfusions, the better the prognosis of the patients. In the course of treatment, with an increase in blood transfusion times, the dilution of coagulation factors may also decrease due to volume expansion, which aggravates coagulation dysfunction of patients and increases the risk of death [28, 29].

There are still significant gaps in this research. One, this is retrospective research, thus blood donations are not at random, and it is more likely to be transfused to patients with severe illnesses. Second, this study covers a large number of diseases, thus making it less contrastive. However, if the types of diseases are limited and the screening conditions are increased, the results may be biased due to an insufficient sample size. The APACHE and SOFA scores do not take into account the incidence of delirium, which is a very common condition in the postoperative ICU and is associated with mortality, thus potentially biasing the results. Future studies should be conducted to further refine treatment protocols to improve short- and long-term outcomes in patients with delirium and to understand risk factors for delirium in the ICU, available monitoring tools, and more [30–32]. In the follow-up, to establish the validity of the findings, a prospective randomized controlled trial with larger sample size is required.

## Conclusion

In conclusion, this study investigated blood transfusion effects on post-major surgery ICU patient mortality. Essential for over 40% of surgical ICU cases, red blood cell transfusions combat anemia and enhance oxygenation. Higher initial ICU APACHE II and SOFA scores correlate with improved survival among major surgery patients. Notably, increased transfusions relate to higher patient mortality. This highlights the critical role of transfusions and underscores the need for refined strategies to enhance outcomes for surgical ICU patients.

## Data Availability

The datasets used and analyzed during the current study are available from the corresponding author on reasonable request.
